# Establishment Performance and Gravel–Soil Characteristics of Planted Saxaul Plantations Across Precipitation Gradients in the Alxa Gobi

**DOI:** 10.3390/plants15142119

**Published:** 2026-07-09

**Authors:** Haibing Wang, Jin Ni, Xue Chen, Hejun Zuo, Zhiying Ning, Xinghua Zhao, Haoqin Yang, Xuan Chen

**Affiliations:** 1 State Key Laboratory of Water Engineering Ecology and Environment in Arid Area, Inner Mongolia Agricultural University, Hohhot 010018, China; 2 College of Desert Control Science and Engineering, Inner Mongolia Agricultural University, Hohhot 010018, China; 3 Inner Mongolia Key Laboratory of Aeolian Physics and Desertification Control Engineering, College of Desert Control Science and Engineering, Inner Mongolia Agricultural University, 29 Erdos East Street, Hohhot 010018, China

**Keywords:** *Haloxylon ammodendron*, afforestation, precipitation gradient, gravel–soil characteristics, Alxa Gobi

## Abstract

In the extremely arid Gobi environment, it remains unclear whether afforestation with saxaul (*Haloxylon ammodendron*) is an effective ecological restoration strategy or whether it may trigger ecological risks under severe water limitation. This study examined saxaul shelterbelts of different ages across precipitation gradients (0–50, 50–100, and 100–150 mm·yr^−1^) in the Alxa Gobi based on 48 plots. It systematically assessed the effects of precipitation on stand growth, soil particle-size distribution, and nutrient dynamics inside and outside plantations, and used partial least squares path modeling (PLS-PM) to analyze the coupling mechanisms among precipitation, soil, and plant growth. The results showed that precipitation was the key factor controlling the survival and growth of *saxaul*, while planting density further regulated survival rates within different precipitation zones. Plant height, crown width, and basal diameter generally showed better performance in higher-precipitation zones, although differences among plantation ages may have been influenced by variation in initial planting density. Among the three precipitation zones, plants in the 100–150 mm zone exhibited the best growth performance. In low-precipitation areas (≤50 mm), the growth of *saxaul* was strongly limited, and afforestation disturbance was associated with disruption of the surface gravel layer, soil coarsening, and inadequate nutrient accumulation. In contrast, in medium- and high-precipitation areas (50–150 mm), saxaul plantations established at appropriate densities are more conducive to the accumulation of fine soil particles and nutrient enrichment. Correlation heatmaps and PLS-PM results further showed that precipitation gradient, soil texture, soil fertility, and plant growth were closely coupled. Moreover, the associations between soil and vegetation variables were stronger inside the shelterbelts than outside the shelterbelts, indicating that more pronounced local soil–vegetation feedbacks may have been formed after the establishment of artificial *Haloxylon ammodendron* stands. Overall, the suitability of saxaul plantations in the Alxa Gobi showed clear precipitation-dependent differentiation, with approximately 50 mm representing a practical lower limit for saxaul plantation establishment. Large-scale saxaul plantations is not recommended in areas with precipitation ≤ 50 mm, where low-disturbance restoration focused on gravel-layer protection should be prioritized; in contrast, areas receiving 50–150 mm precipitation are more suitable for plantation establishment under appropriate density control. These findings provide a scientific basis for sustainable afforestation, regional allocation, and low-disturbance management in extremely arid Gobi regions under the principle of matching vegetation restoration to water availability and site conditions.

## 1. Introduction

Against the backdrop of global climate change, the stability of ecosystems in arid and semi-arid regions faces severe challenges [1]. The Gobi Desert, located in the core of the Central Asian arid zone, is experiencing dual pressures from climate warming and human activities that undermine its fragile ecological balance [2]. As a typical extreme ecosystem in the arid inland regions of Asia, the Gobi Desert is not only an important desert landscape type but also one of the major dust source regions in northern China [3]. Characterized by scarce precipitation, intense evaporation, infertile soils, and significant wind erosion, both vegetation establishment and surface stability are strongly constrained by water availability, and the region is highly sensitive to external disturbances [2,4]. Against this backdrop, saxaul (*Haloxylon ammodendron*) has been widely used for windbreaks, sand fixation, and vegetation restoration in the Gobi Desert region of Northwest China due to its strong tolerance to drought, salinity, alkalinity, and wind erosion [5]. However, in extremely arid Gobi landscapes, it remains unclear whether saxaul plantations can achieve stable establishment and generate gravel–soil feedbacks that are conducive to surface stability. Clarifying how precipitation controls plantation establishment performance and associated soil feedback processes has become a key scientific issue in vegetation restoration research in arid regions.

Unlike typical sandy ecosystems, the surface of the Gobi is extensively covered by a gravel mantle formed through long-term aeolian sorting, physical weathering, and the rearrangement of surface materials [6]. This unique surface structure not only enhances surface resistance to erosion and reduces the loss of fine particles, but also retains dew to some extent, inhibits evaporation, and regulates the hydrothermal conditions of shallow soils, thereby maintaining the relative stability of the near-surface system under extremely arid conditions [7]. The Gobi Desert not only preserves a wealth of paleoenvironmental information but also sustains fragile desert biodiversity, represented by extreme xerophytic plants and soil microorganisms [8]. Through synergistic interactions, these biological components maintain material cycling and energy flow under extreme environmental conditions [9,10]. However, human activities such as overgrazing, mineral extraction, and inappropriate vegetation restoration can disrupt the integrity of the gravel layer, weaken surface stability, and further trigger a chain of processes including increased wind erosion, fine particle loss, and soil degradation [11]. Therefore, when carrying out afforestation in the Gobi region, attention should be paid not only to vegetation restoration itself, but also to its potential impact on the stability of the gravel layer and the near-surface environment.

Among the various measures for artificial vegetation restoration in the Gobi region, saxaul is one of the most widely used and representative extreme xerophytic shrubs [12]. *saxaul* is a typical C4 desert shrub belonging to the genus *Haloxylon* in the Chenopodiaceae family [13]. It is naturally distributed in the arid regions of Central Asia and the desert areas of northwestern China, primarily found in Xinjiang, Gansu, Qinghai, and western Inner Mongolia [14,15], and typically grows along the margins of the Gobi Desert or in gravelly plain areas where precipitation is relatively higher [16]. In recent years, driven by government policies, large-scale plantations of artificial saxaul plantations have been established in Gobi regions such as western Inner Mongolia, and have become an important practical approach to desertification control and ecological restoration in areas like Alxa [17]. Existing research indicates that saxaul planting can improve soil structure, promote nutrient accumulation, and enhance surface stability in some areas; however, in regions with extreme water scarcity, high-density or improperly configured plantations may lead to plant decline, soil moisture depletion, and even damage to the surface gravel layer due to sustained competition for resources, thereby producing negative ecological effects [18,19]. Furthermore, in the Gobi region, where site conditions are complex and management is relatively extensive, artificial saxaul plantations may also face issues such as low seedling survival rates, stand degradation, and landscape fragmentation [20]. This indicates that saxaul plantations does not necessarily yield sustained and stable ecological benefits, and its ecological effects are highly dependent on environmental conditions.

Among the many factors influencing the restoration success of artificial saxaul plantations, precipitation is one of the most fundamental and critical environmental constraints [21]. As the primary limiting factor in arid ecosystems, precipitation not only affects saxaul seed germination, seedling establishment, and the growth of mature plants, but also determines their long-term survival and population sustainability [19]. However, existing studies have largely focused on single precipitation conditions or short-term observations, and there remains a lack of systematic comparisons of the growth characteristics of artificial saxaul plantations under different precipitation gradients. In particular, there is insufficient understanding of the synergistic response patterns of indicators such as survival rate, plant height, crown width, and basal diameter. At the same time, the feedback between vegetation and soil is a key mechanism for ecosystem stability in arid regions [22]. The growth of saxaul plantations influences soil particle size distribution and nutrient characteristics through processes such as litter input, while changes in the soil, in turn, affect vegetation growth [23]. Although existing studies have focused on the soil-improving effects of saxaul plantations, current research has paid little attention to the impact of saxaul plantations on gravel layer integrity and soil nutrient dynamics under different precipitation gradients in the Gobi region, making it difficult to elucidate the coupling mechanisms among precipitation, vegetation, and soil. It is worth noting that existing research on artificial saxaul plantations has primarily focused on habitats such as oasis margins and sandy areas at the edges of deserts. While significant insights have been gained regarding population structure [24], soil water use [12], afforestation patterns [25], protective measures [26], and degradation processes [27], systematic studies across precipitation gradients in genuine Gobi environments, characterized by gravel cover, the absence of groundwater recharge, and extreme wind erosion, remain scarce. Currently, systematic research on precipitation thresholds, the growth status of saxaul plantations, gravel layer integrity, changes in soil nutrients, and the cost-effectiveness of afforestation in Gobi regions remains clearly inadequate, resulting in a lack of adequate theoretical basis for afforestation decisions in these areas.

Against this background, this study focuses on artificially planted saxaul plantations in the Alxa Gobi Desert region under varying precipitation gradients. By incorporating plots of different plantation ages, the study systematically quantifies the effects of afforestation on vegetation growth, the stability of the surface gravel layer, and changes in soil properties. The study aims to address the following scientific questions: (1) How do varying precipitation gradients in the Gobi Desert region affect the growth of saxaul plantations of different plantation ages? (2) How does afforestation in the Gobi Desert affect the gravel layer? (3) What is the relationship between saxaul growth performance and soil property changes across precipitation gradients? (4) How should afforestation strategies for saxaul plantations be optimized based on precipitation conditions? Furthermore, the survival rate, growth status, afforestation costs, and sand control effectiveness of saxaul plantations in the Gobi region ultimately point to a more practical question: Is the Gobi region suitable for large-scale promotion of saxaul plantations, and under what precipitation and site conditions would implementation be more sustainable? Based on these scientific questions, we hypothesized that increasing precipitation would promote the establishment and growth of saxaul plantations in the Alxa Gobi. We further expected higher precipitation to mitigate adverse impacts on the gravel layer and soil properties, thereby enhancing fine-particle and nutrient accumulation and strengthening soil–vegetation coupling within plantations. Therefore, saxaul afforestation in Gobi regions should be optimized according to precipitation conditions, gravel-layer stability, and initial planting density. The research findings will elucidate the mechanism by which precipitation acts as a key driving factor, providing scientific support for implementing the principle of “site-specific afforestation” in the Gobi region. In particular, they will offer a theoretical basis for determining appropriate planting scales and mitigating ecological risks in the extremely arid Gobi region.

## 2. Methods

### 2.1. Overview of the Study Area

**Figure 1:**
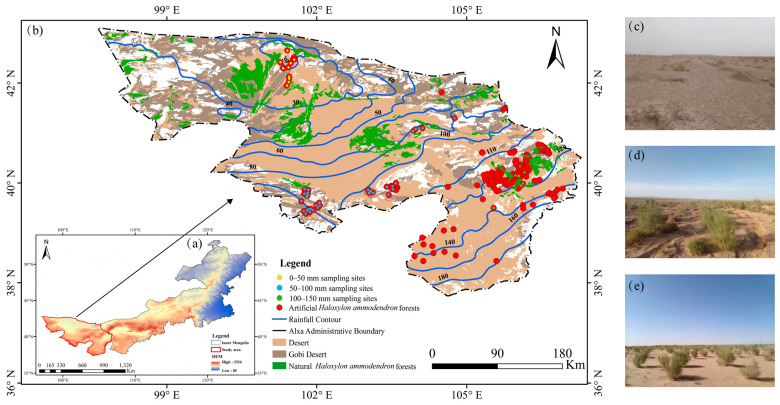
Overview of the study area and field photographs of *saxaul* in different precipitation zones. Note: (**a**) Administrative map of the Inner Mongolia Autonomous Region; (**b**) Distribution map of *saxaul* in the Alxa Region; (**c**) Field view of Zone I (0–50 mm); (**d**) Field view of Zone II (50–100 mm); (**e**) Field view of Zone III (100–150 mm).

The study area is located in the Gobi Desert of Alxa League, Inner Mongolia Autonomous Region, China, between the Yellow River and the Helan Mountains (Figure 1). The region has a typical arid continental climate, with an average annual temperature of 6–8 °C, annual evaporation ranging from 2000 to 3900 mm, and annual precipitation of only 32.8–208.1 mm. The prevailing wind direction in the study area is northwesterly, resulting in severe wind erosion.

This study covers three precipitation gradients in the Alxa Gobi region: Zone I (0–50 mm·yr^−1^), Zone II (50–100 mm·yr^−1^), and Zone III (100–150 mm·yr^−1^), with the corresponding climatic characteristics shown in Table 1. To ensure comparability across different precipitation zones, plots were selected according to the following criteria: the parent material of the soil was alluvial–proluvial gravelly soil; the terrain was flat, with a slope < 5°; there was no groundwater recharge or the water table was >10 m; and prior to afforestation, all sites were covered by native Gobi vegetation and had not undergone agricultural cultivation or mining disturbance. These control conditions minimized the influence of environmental factors other than precipitation. The terrain slopes from east to west, featuring alternating desert and Gobi landscapes. The predominant soil type is gravelly soil with high gravel coverage. The study area is dominated by artificially planted stands of *Haloxylon ammodendron*, with associated species including *Sarcozygium xanthoxylum*, *Reaumuria soongorica*, and *Calligonum mongolicum*. Naturally regenerating herbaceous plants include *Agriophyllum pungens* and *Peganum harmala*, among others.

### 2.2. Data Collection and Measurement

#### 2.2.1. Plot Selection

Through field surveys, we selected a total of 48 plots of artificially planted *saxaul* plantations in the Gobi Desert across three precipitation zones for investigation (as shown in Figure 1). Each plot measured 100 m × 100 m, and within each plot, three 10 m × 10 m subplots were established for vegetation surveys. Plot selection criteria: clearly defined plantation age with traceable afforestation records; known initial planting density; no artificial irrigation or replanting; and a distance of >500 m between plots to ensure independence.

#### 2.2.2. Vegetation Survey

A survey was conducted on the saxaul plantation establishment process and the growth status of saxaul shelterbelts within the sample plots. Key parameters included planting density, survival rate, plantation age, plant height, crown width, and basal diameter. Survival rate was calculated as follows: Survival rate (%) = Number of surviving plants/Initial planting density × 100%. Plant height was measured using a measuring rod; crown width was measured using the cross method; and basal diameter was measured at 5 cm above the stem base using a digital vernier caliper, with an accuracy of ±0.1 mm. It should be noted that the sampled plantations in this study were selected from the “100 Million Saxaul” project initiated by the Society of Entrepreneurs and Ecology (SEE) Foundation in the Alxa region, including saxaul plantations supported through Ant Forest. According to the project afforestation records and official seedling specifications, the planted Haloxylon ammodendron seedlings were nursery-grown seedlings with relatively uniform specifications, with an initial seedling height of approximately 40 cm and a root length of approximately 20 cm. Because plot-specific baseline height records for individual seedlings before planting were not available, plant height in this study was mainly used to characterize the standing growth status of surviving plantations at the time of field investigation, rather than the absolute height increment after planting.

#### 2.2.3. Soil Sampling

Within each plot, soil samples were collected both within the saxaul plantation (20–50 cm from the base of the plants) and outside the plantation (on open, bare ground more than 10 m from the plantation edge). Topsoil (0–2 cm) was collected using the five-point sampling method and mixed into a single sample; profile soils (0–100 cm) were sampled in layers at 0–10, 10–20, 20–30, 30–40, 40–50, 50–60, 60–70, 70–80, 80–90, and 90–100 cm; three replicates were collected from each layer and then mixed. During collection, impurities such as plant roots and debris were removed, and the samples were placed in self-sealing bags, labeled, and returned to the laboratory for analysis. A total of 96 surface soil samples (48 plots × inside/outside the plantation) and 960 profile soil samples (48 plots × inside/outside the plantation × 10 soil layers) were collected for this study.

#### 2.2.4. Soil Sample Analysis

After collection, the soil samples were air-dried and impurities were removed, then graded by particle size using a combination of sieving and a laser particle size analyzer. Particle size classification followed the U.S. standard: silt (0.002–0.05 mm), very fine sand (0.05–0.10 mm), fine sand (0.10–0.25 mm), medium sand (0.25–0.50 mm), coarse sand (0.50–2.00 mm), and gravel (>2 mm). Conventional soil chemical analysis methods were used to determine total nitrogen (TN), total phosphorus (TP), total potassium (TK), alkali-hydrolyzable nitrogen (AN), available phosphorus (AP), and available potassium (AK) [28,29]. The specific determination methods were as follows: TN was determined using the Kjeldahl method; TP using the molybdenum–antimony colorimetric method; TK using the flame photometric method; AN using the alkaline hydrolysis diffusion method; AP using the sodium bicarbonate extraction–molybdenum–antimony colorimetric method; and AK using the ammonium acetate extraction–flame photometric method.

### 2.3. Data Analysis

All statistical analyses were performed using R software version 4.2.2. Because the sampled plantations were selected from existing afforestation projects, stand–age combinations were incomplete across precipitation zones, and initial planting density varied among age classes. Therefore, the dataset was not treated as a full precipitation × plantation age controlled experimental design. To avoid overinterpreting stand–age effects, this study mainly compared the standing growth status of surviving saxaul shelterbelts across precipitation zones and plantation development stages, while discussing the potential influence of initial planting density on survival rate and growth performance. Age-related results were interpreted mainly as descriptive comparisons rather than as strict temporal dynamics. One-way analysis of variance (ANOVA) was used to compare differences in growth indices and soil properties across different precipitation zones and plantation ages, with Tukey’s post hoc test (α = 0.05) applied for multiple comparisons. Data were presented as “mean ± standard deviation.” The bivariate correlations among precipitation gradient, plant growth indicators, soil texture PC1, and soil nutrient indicators were evaluated using Spearman correlation analysis, and correlation heatmaps were generated separately for inside-shelterbelt and outside-shelterbelt samples. Soil texture PC1 was derived from principal component analysis of the soil particle-size fractions and was used to comprehensively represent variations in soil particle-size composition. Partial least squares path modeling (PLS-PM) was used to analyze the coupling relationships among precipitation gradients, soil texture, soil fertility, and plant growth. The significance of model path coefficients was tested using the Bootstrap method, with a 95% confidence interval. Separate models were constructed for inside- and outside-plantation areas for comparison.

## 3. Results

### 3.1. Growth Characteristics of Saxaul Plantations in Different Precipitation Zones

This study investigated the planting density and survival rate of artificial saxaul plantations under different precipitation gradients (0–50 mm, 50–100 mm, 100–150 mm) and different plantation ages (2–6 years) (Table 2). The results showed that the planting density and survival rate of saxaul plantations exhibit distinct patterns of variation with precipitation and plantation age. Overall, the survival rate of saxaul plantations showed an upward trend with increasing precipitation. In Zone I (0–50 mm), the average survival rate of 2-year-old plantations was only 27%, while that of 6-year-old plantations was 54%. In Zone II (50–100 mm), the average survival rates of 3-year-old, 4-year-old, and 6-year-old plantations were 54%, 81%, and 54%, respectively. In Zone III (100–150 mm), the average survival rates for 4-year-old and 6-year-old plantations were 51% and 68%, respectively. It is worth noting that survival rates within the same precipitation zone were closely related to initial planting density. Overall, within the same precipitation zone, plots with lower initial planting densities typically exhibit higher survival rates, while those with higher initial planting densities have relatively lower survival rates (Table 2).

**Figure 2:**
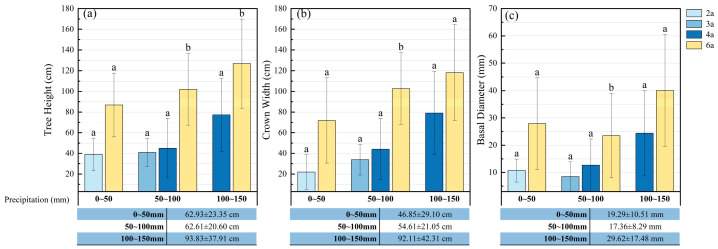
Variation in the height (**a**), crown width (**b**), and basal diameter (**c**) of *saxaul* under different precipitation gradients and plantation ages. Note: Different lowercase letters indicate significant differences among plantation ages within the same precipitation zone for the same growth parameter (*p* < 0.05). Error bars represent standard deviations. (**a**), (**b**) and (**c**) represent plant height, crown width, and basal diameter, respectively; 2a, 3a, 4a, and 6a denote saxaul shelterbelts with plantation ages of 2, 3, 4, and 6 years, respectively; the bottom of each figure shows the mean values of plant height, basal diameter, and crown width under different precipitation conditions, with data presented as “mean ± standard deviation”.

Overall, plant height, crown width, and basal diameter increased with rising rainfall, and differences were also observed among different plantation ages (Figure 2). Regarding plant height, the average height of plants in Zone III (100–150 mm) reached 93.83 ± 37.91 cm, representing increases of 49.8% and 49.0% compared to Zones I (0–50 mm) and II (50–100 mm), respectively. A similar trend was observed for crown width: the mean crown width in Zone III (100–150 mm) was 92.11 ± 42.31 cm, representing increases of 68.6% and 96.6% compared to Zones I (0–50 mm) and II (50–100 mm), respectively. This indicates that adequate precipitation significantly promotes vertical growth, confirming the critical role of water supply in stem thickening. It is worth noting that the basal diameter was also highest in Zone III (100–150 mm) (29.62 ± 17.48 mm), while no significant differences were observed between Zone I (0–50 mm) and Zone II (50–100 mm). Overall, as rainfall increased, plant height, crown width, and basal diameter generally showed an upward trend. As plantation age increased, these trends remained consistent, and the growth condition of *saxaul* gradually improved.

### 3.2. Changes in Gravel-Layer Characteristics in Gobi Afforestation Sites Under Different Precipitation Gradients

#### 3.2.1. Particle Size Characteristics of the Surface Soil

**Figure 3:**
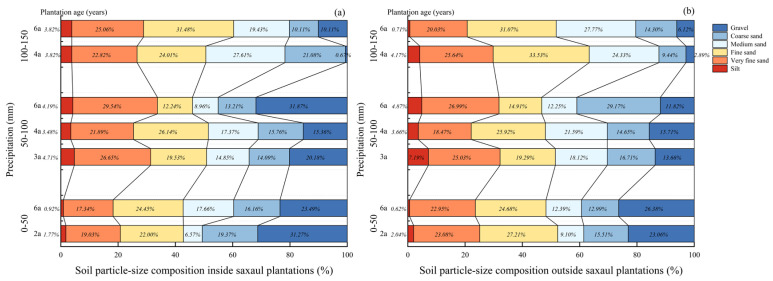
Soil Particle Size Characteristics Inside and Outside *saxaul* plantation under Different Precipitation Gradients: (**a**) inside plantations and (**b**) outside plantations.

In Zone I (0–50 mm), the surface soil was dominated by coarse particles; in the 2-year-old stand, gravel and coarse sand accounted for 50.49% of the total, which is significantly higher than that outside the stand. As plantation age increased from 2 to 6 years, the proportion of gravel and coarse sand decreased within the stand, while the proportions of fine sand and medium sand increased. Zone II (50–100 mm) exhibited distinct transitional characteristics in particle size distribution, with relatively high proportions of very fine sand and fine sand; however, some stands still maintained high proportions of gravel or coarse sand. In Zone III (100–150 mm), the proportion of fine particles increased significantly. In the 4-year-old and 6-year-old plantations within the stand, medium sand (27.61%) and fine sand (31.48%) were the dominant fractions, respectively. The 6-year-old saxaul stand showed a significant increase in fine-particle content, with silt, very fine sand, and fine sand accounting for 60.36%. Under conditions of higher precipitation, increasing plantation age was more conducive to the accumulation of fine-particle content and the optimization of surface soil structure (Figure 3).

#### 3.2.2. Vertical Distribution of Soil Particle Size in Soil Profiles

**Figure 4:**
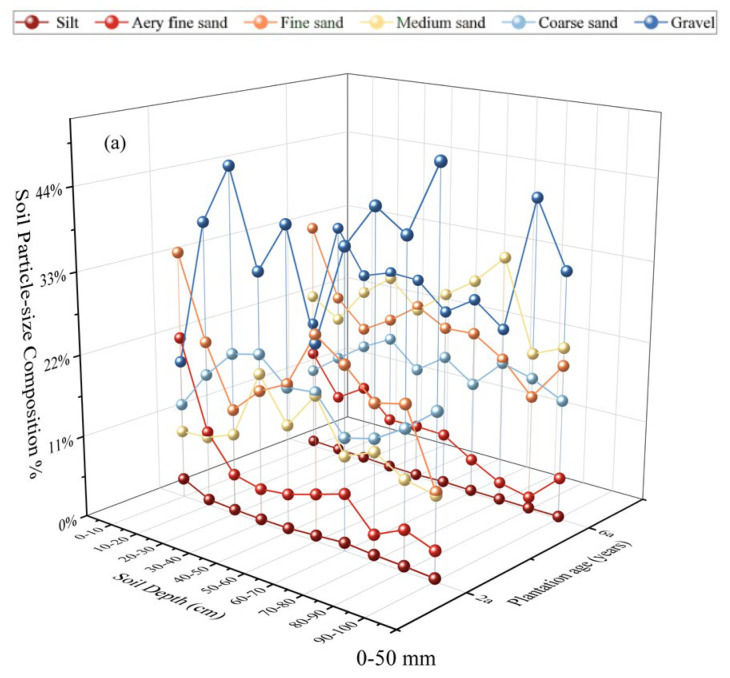

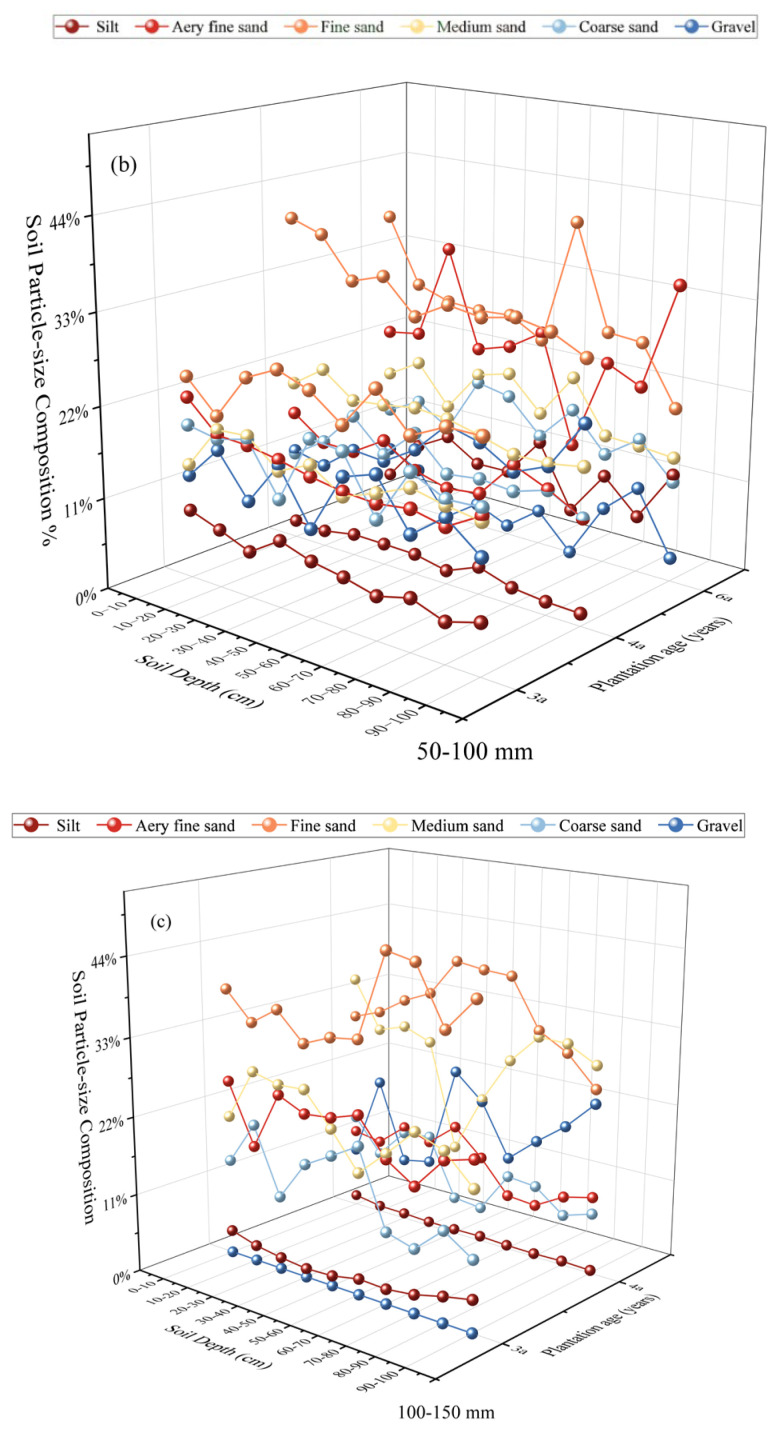
Soil Particle Size Characteristics in *saxaul* Plantation Profiles under Different Precipitation Gradients: (**a**) 0–50 mm·yr^−1^, (**b**) 50–100 mm·yr^−1^, and (**c**) 100–150 mm·yr^−1^.

In Zone I (0–50 mm), as soil depth increases, the content of silt and very fine sand decreases significantly, while the proportion of coarse sand and gravel gradually increases. Fine and medium sand are primarily concentrated in the 0–20 cm soil layer, while their content fluctuates considerably in deeper soil layers. Compared to the 2-year-old plantations, the fluctuations in the content of each grain size class across different soil layers are relatively smaller in the 6-year-old plantations. In Zone I, the 0–10 cm soil layer showed lower gravel content and an increase in sand particles, consistent with observations that afforestation has disrupted the surface gravel layer; in the 10–30 cm soil layer, the content of fine particles such as silt and very fine sand decreased sharply, while the content of coarse sand and gravel rose rapidly. In Zone II (50–100 mm), the vertical fluctuations in the content of silt, very fine sand, and fine sand in the soil of 3-year-old saxaul plantations were relatively smaller than those in Zone I. The contents of very fine sand and fine sand were also relatively stable. Under these precipitation conditions, saxaul plantations were better able to regulate soil particle distribution and improve soil quality. In Zone III (100–150 mm), overall, as soil depth increases, the silt content showed a trend of first decreasing and then fluctuating; the very fine sand content showed a decreasing trend but remained relatively stable; the fine sand content fluctuates, exhibiting different patterns across different plantation ages and depths; the content of medium and coarse sand fluctuated significantly; the decrease in gravel content within Zone III plantations was relatively small, indicating that saxaul plantations in the moderate-precipitation zone cause minimal surface disturbance. Gravel content was 0 throughout the entire profile at the 4-year-old stand, whereas at the 6-year-old stand, it was present in the deep soil layers and exhibited fluctuating changes (Figure 4).

### 3.3. Soil Nutrient Characteristics in Gobi Afforestation Under Different Precipitation Gradients

#### 3.3.1. Nutrient Characteristics of the Surface Soil

**Figure 5:**
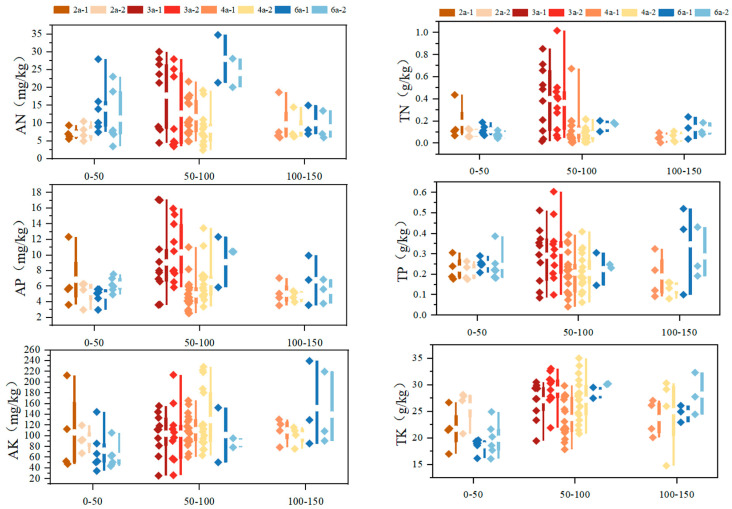
Soil Nutrient Characteristics in *saxaul* plantation under Different Precipitation Gradients. Note: AN, AP, AK, TN, TP, and TK represent alkali-hydrolyzable nitrogen, available phosphorus, available potassium, total nitrogen, total phosphorus, and total potassium, respectively. 2a, 3a, 4a, and 6a represent saxaul shelterbelts with plantation ages of 2, 3, 4, and 6 years, respectively; -1 indicates sampling points located inside the stand, and -2 indicates sampling points located outside the stand. For example, “2a-1” denotes an in-shelterbelt sampling point in a 2-year-old saxaul stand, and so on.

As rainfall increased from 0–50 mm to 100–150 mm, overall, the AN, AP, and AK contents in both inside- and outside-plantation soils first increased and then decreased (Figure 5). In Zone II, the topsoil nutrient content in the saxaul plantation was the highest, with AN, AP, and AK reaching their highest levels, particularly evident in inside-plantation soils, and the difference between inside- and outside-plantation soils gradually widened. In Zone II, the AN, AP, and AK concentrations in the inside-plantation soils of the 4-year-old plantations were 42.3%, 38.7%, and 31.2% higher, respectively, than those in the outside-plantation soils (*p* < 0.01). In Zone III, soil nutrient concentrations showed some recovery but exhibited significant fluctuations. In Zone I, both the overall nutrient levels and the degree of differentiation were low, with no significant differences between inside- and outside-plantation soils, which may be due to water limitation. However, the levels of available soil nutrients inside the plantation were not significantly higher than those outside the plantation. Total nitrogen (TN) and total phosphorus (TP) in inside-plantation soils were approximately 30% and 25% higher, respectively, than in outside-plantation soils, while the difference in total potassium (TK) was relatively small. Furthermore, as plantation age increased, the gap between inside- and outside-plantation soils gradually widened: differences were not significant in 2-year-old plantations, whereas the nutrient advantage inside the plantation was most pronounced in 6-year-old plantations.

#### 3.3.2. Vertical Distribution of Soil Nutrients in Soil Profiles

**Figure 6:**
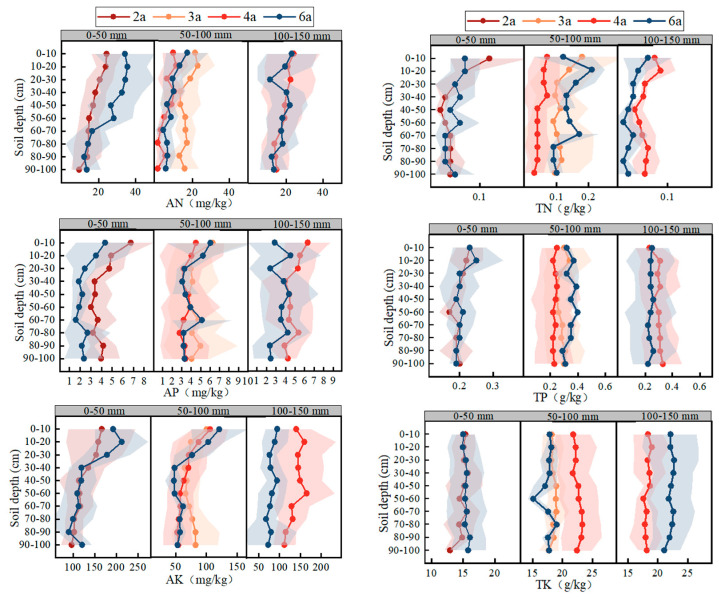
Soil Nutrient Characteristics in *saxaul* Plantation Profiles under Different Precipitation Gradients. Note: AN, AP, AK, TN, TP, and TK represent alkali-hydrolyzable nitrogen, available phosphorus, available potassium, total nitrogen, total phosphorus, and total potassium, respectively. 2a, 3a, 4a, and 6a represent saxaul plantations with plantation ages of 2, 3, 4, and 6 years, respectively; the shaded areas indicate the standard deviation.

As shown in Figure 6, the nutrient content in the 0–100 cm soil profile of saxaul plantations exhibits distinct trends across different precipitation gradients. Overall, nutrient levels increase slightly with depth across different precipitation zones, but the magnitude of this increase is small, and nutrient levels remain generally low, making it difficult for nutrients to accumulate effectively. In Zone I (0–50 mm), soil nutrient concentrations were lowest in the 0–10 cm layer, and while there was a slight increase in the deeper soil layers (10–100 cm), overall levels remained low. Even in 6-year-old plantations, nutrient levels in both the surface and deep soil layers remained low. In Zone II (50–100 mm), soil nutrient levels increased significantly, particularly in the 0–10 cm surface layer. As plantation age increased, soil nutrient levels rose markedly, especially in 6-year-old plantations. In Zone II, the concentrations of TN, TP, AN, AP, and AK in the 0–10 cm soil layer of 6-year-old plantations were 35.6%, 28.3%, 52.1%, 44.7%, and 38.4% higher, respectively, than those in 2-year-old plantations.

In Zone III (100–150 mm), AK and TK levels in the deep soil layer (10–100 cm) exhibited significant fluctuations. Although TN and TP levels were relatively high in the 0–10 cm surface soil layer, nutrient levels in the deep soil layer were unstable due to nutrient leaching caused by excessive precipitation. Consequently, the effects of plantation age were less pronounced in the deep soil layer (10–100 cm) than in the moderate precipitation zone.

### 3.4. Relationship Between Vegetation and Soil in Gobi Afforestation Under Different Precipitation Gradients

To further clarify the association characteristics among saxaul growth, soil texture, and soil fertility under different rainfall gradients, correlation analysis was conducted separately for inside-shelterbelt and outside-shelterbelt samples. In addition, partial least squares path modeling (PLS-PM) was used to analyze the statistical coupling relationships among rainfall gradient, soil texture, soil fertility, and plant growth. The correlation heatmaps were used to characterize the bivariate correlation structure among variables, while PLS-PM was further used to compare the pathway relationships among different variable groups inside and outside the shelterbelts.

**Figure 7:**
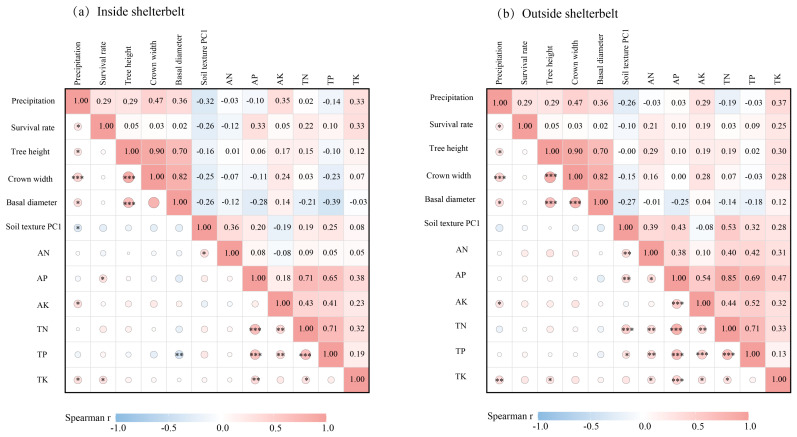
Spearman correlation heatmaps of soil–vegetation variables inside and outside saxaul shelterbelts under different rainfall gradients. Note: (**a**) Inside-shelterbelt samples; (**b**) outside-shelterbelt samples. Colors indicate the magnitude of Spearman correlation coefficients, with red representing positive correlations and blue representing negative correlations. The numbers in the upper triangle represent correlation coefficients, and the circle size in the lower triangle indicates correlation strength. *, **, and *** indicate significance levels of *p* < 0.05, *p* < 0.01, and *p* < 0.001, respectively. Soil texture PC1 represents the first principal component of soil particle-size composition. AN, AP, AK, TN, TP, and TK represent alkali-hydrolyzable nitrogen, available phosphorus, available potassium, total nitrogen, total phosphorus, and total potassium, respectively.

The correlation heatmaps showed that the correlation structures among variables in the inside-shelterbelt and outside-shelterbelt samples were partly consistent but also showed certain differences (Figure 7). Overall, rainfall gradient was positively correlated with survival rate, plant height, crown width, and basal diameter, and strong correlations were observed among plant height, crown width, and basal diameter, indicating a consistent response of saxaul growth indicators to increasing precipitation. In the inside-shelterbelt samples, soil texture PC1 showed weak negative correlations with rainfall gradient and some growth indicators, whereas nutrient indicators such as AP, AK, TN, and TP showed relatively clear positive correlations, suggesting a certain degree of coordinated variation between soil particle-size composition and nutrient status inside the shelterbelts. In the outside-shelterbelt samples, correlations among soil nutrient indicators were more concentrated. Soil texture PC1 was positively correlated with AN, AP, TN, TP, and other indicators, but its associations with plant growth indicators were generally weak. This indicates that outside-shelterbelt soils mainly reflect background soil differences along the rainfall gradient, whereas in the inside-shelterbelt samples, the relationships among plant growth, soil particle-size composition, and nutrient status better reflect the local soil–vegetation coupling characteristics after the establishment of artificial saxaul shelterbelts.

**Figure 8:**
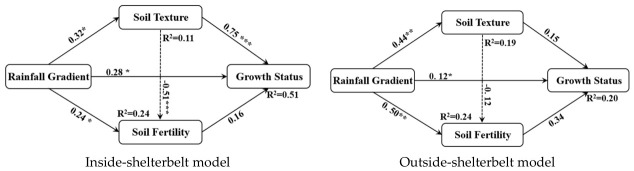
Coupling relationships among rainfall gradient, soil texture, soil fertility, and saxaul growth status inside and outside stands revealed by partial least squares path modeling (PLS-PM). Note: Values on the paths represent standardized path coefficients. *, **, and *** indicate significance at *p* < 0.05, *p* < 0.01, and *p* < 0.001, respectively, based on bootstrap tests. Line types: solid lines indicate positive correlation paths, and dashed lines indicate negative correlation paths.

The results of the partial least squares path modeling (PLS-PM) showed clear differences between inside-shelterbelt and outside-shelterbelt samples in the coupling relationships among rainfall gradient, soil texture, soil fertility, and plant growth (Figure 8). Inside the shelterbelts, rainfall gradient showed significant positive path relationships with soil texture (path coefficient = 0.32, *p* < 0.05) and soil fertility (path coefficient = 0.24, *p* < 0.05), and also showed a significant positive path relationship with saxaul growth status (path coefficient = 0.28, *p* < 0.05). The path coefficient between soil texture and saxaul growth status was the highest (path coefficient = 0.75, *p* < 0.001). Soil texture also showed a significant negative path relationship with soil fertility (path coefficient = −0.51, *p* < 0.001), whereas the path relationship between soil fertility and plant growth was not significant (*p* > 0.05). These results indicate that, in the extremely arid Gobi environment, differences in plant growth may be more closely associated with rainfall background and soil physical structure, while the direct explanatory role of nutrient conditions is relatively weak.

Outside the shelterbelts, rainfall gradient also showed significant positive path relationships with soil texture and soil fertility, with path coefficients of 0.44 and 0.50, respectively (*p* < 0.01). The path coefficient between rainfall gradient and saxaul growth status was relatively low (path coefficient = 0.12, *p* < 0.01). Meanwhile, the path relationships between soil texture and saxaul growth status, between soil fertility and saxaul growth status, and between soil texture and soil fertility were not significant (*p* > 0.05). This suggests that although outside-shelterbelt soil properties can reflect background differences along the rainfall gradient, their coupling relationship with actual saxaul growth status is weaker than that inside the shelterbelts.

In terms of model explanatory power, the inside-shelterbelt model explained plant growth much better (R^2^ = 0.51) than the outside-shelterbelt model (R^2^ = 0.20), indicating a closer statistical coupling between soil variables and saxaul growth inside the shelterbelts. The model fit indices showed that the GoF values were 0.62 for the inside-shelterbelt model and 0.51 for the outside-shelterbelt model, both within an acceptable range. Overall, the correlation heatmaps in Figure 7 and the PLS-PM results in Figure 8 indicate that saxaul growth in the Alxa Gobi is not only associated with rainfall gradient, but also closely related to soil particle-size composition, nutrient status, and changes in the local inside-shelterbelt environment.

## 4. Discussion

### 4.1. Establishment Success and Suitability Variation of Saxaul Plantations Under Precipitation Constraints

Precipitation has a significant impact on the survival rate of *saxaul* [29]. This study indicates that the survival rate of *saxaul* in Zone I (0–50 mm) is significantly constrained, suggesting that extremely low precipitation conditions significantly limit seedling establishment and early survival [30], indicating that water scarcity is a key limiting factor for survival. Although the survival rate of 6-year-old plantations in Zone I was higher than that of 2-year-old plantations, the overall level remained significantly lower than that in Zones II and III, indicating that, under persistent drought conditions, increasing plantation age does not fundamentally alter the limiting effect of water stress on stand survival. It is worth noting that survival rate is jointly regulated by precipitation conditions and initial planting density. In Zone II (50–100 mm), the survival rate of 4-year-old plantations reached 81%, which was significantly higher than that of 3-year-old and 6-year-old plantations in the same zone, and also higher than that of stands of the same age in high-precipitation zones (the survival rate of 4-year-old plantations in Zone III was only 51%). Combined with Table 2, it can be seen that the initial planting density of 4-year-old plantations in Zone II was relatively low, while that of 6-year-old plantations was the highest. This suggests that under moderate precipitation conditions, an appropriate initial planting density may be a stronger determinant of survival rates than plantation age itself. Excessively high density intensifies competition among individuals for limited water resources, thereby undermining the survival advantage brought about by improved precipitation conditions. Therefore, in the 50–100 mm precipitation zone, higher precipitation is not necessarily better; rather, optimal establishment results are more likely to be achieved when moderate precipitation is matched with an appropriate planting density. Similarly, in Zone III (100–150 mm), the survival rate of 6-year-old plantations (68%) was higher than that of 4-year-old plantations (51%), even though the initial planting density was lower than that of the 4-year-old plantations. This further illustrates that while higher precipitation conditions help alleviate water stress during long-term growth, stand survival rates are still significantly influenced by differences in initial planting density. In other words, precipitation determines the overall ecological context for the success or failure of saxaul plantations, while initial planting density moderates the actual survival rate differences within the same precipitation zone; when initial planting density is too high, even in a relatively suitable precipitation zone, survival rates may decline due to increased intraspecific competition.

Under different precipitation gradients, the plant height, crown width, and basal diameter of *saxaul* all increased significantly with rising rainfall and plantation age, consistent with the general principle that vegetation growth in arid regions is limited by water availability [31]. Particularly in Zone III, which has higher annual precipitation, *saxaul* exhibited superior growth, further confirming the critical role of water in biomass accumulation and structural optimization. It is worth noting that there was no significant difference in basal diameter between Zone II and Zone I. This may be because limited water is prioritized for maintaining basic growth and survival rather than for energy-intensive secondary growth processes such as woody tissue thickening [13]. Comparing the results of this study with precipitation thresholds established for vegetation in global arid regions holds significant theoretical importance. Noy-Meir [32] defined arid regions as areas with an annual average precipitation of <250 mm and high precipitation variability. However, this study found that the lower limit for suitable saxaul plantations in the Gobi region (50 mm) is far below this classic threshold, reflecting the unique adaptability of saxaul as an extreme xerophyte. At the same time, the results of this study echo the criticism by Cao et al. [33] regarding excessive afforestation in China’s arid regions: in areas with precipitation below 50 mm, the long-term survival rate of planted vegetation is extremely low; large-scale afforestation not only fails to achieve the expected ecological benefits but may also lead to resource waste and ecological risks.

Further analysis of growth indicators for *saxaul* under different site conditions (Table 3) reveals that the survival rate and growth performance of *saxaul* in Gobi habitats are generally lower than those in salt lake, saline, and fixed or semi-fixed dune habitats. This indicates that *saxaul* is more likely to establish a sustained and stable growth advantage in sites with relatively better moisture conditions, higher fine-particle content, or more stable groundwater recharge. Conversely, in Gobi regions, scarce precipitation, intense wind erosion, the susceptibility of gravel layers to disturbance from land preparation, and the difficulty in achieving stable accumulation of fine particles and nutrients result in higher establishment risks and lower growth efficiency for saxaul plantations. Based on the above evidence, this study explicitly concludes that Gobi regions, particularly extremely arid Gobi areas with annual precipitation ≤ 50 mm, are not suitable for indiscriminate large-scale promotion of saxaul plantations.

It should be noted that the sampled plots in this study were derived from actual afforestation projects, and the initial planting densities were not fully consistent among stands of different ages. Therefore, plantation age effects may be confounded with planting density effects. In this study, plantation age was not interpreted as an independent driving factor, but rather as a descriptive variable representing stand development stage, and was considered together with initial planting density to explain differences in saxaul survival rate and growth performance. Future studies based on the same initial planting density or long-term fixed-plot monitoring are needed to further distinguish plantation age effects from density effects.

### 4.2. Synergistic Response of the Gravel Layer and Soil Nutrients

The regulatory effect of vegetation restoration in arid regions on soil particle size structure is significantly influenced by precipitation conditions. Afforestation activities may either disrupt the surface gravel layer or, under moderate moisture conditions, promote the accumulation of fine particles and structural optimization. This study found that in Zone I (0–50 mm), the proportion of coarse sand in the inside-plantation soil was significantly higher than that outside the plantation during the early stages of saxaul plantation establishment (Figure 3), indicating that artificial land preparation disturbances may have disrupted the protective surface gravel layer, and that stands with low survival rates (≤0.54) (Table 2) failed to effectively suppress subsequent wind erosion-induced coarsening. This result is consistent with the observations of Yao et al. [37], namely that afforestation disturbances in arid regions can compromise the physical integrity of the surface gravel layer. In contrast, in Zone II (50–100 mm) and Zone III (100–150 mm), saxaul plantations significantly reduced the proportion of coarse sand, with the most significant reduction observed in 6-year-old plantations in Zone III. This indicates that saxaul plantations in areas with moderate precipitation cause minimal surface disturbance and can promote the accumulation of fine particles through canopy interception and root stabilization.

At the profile scale, the 010 cm soil layer in Zone I had a low gravel content and an increased sand content, consistent with observations that afforestation disrupts the surface gravel layer [37]. The topsoil still retained a certain amount of fine particles, but in the 10–30 cm soil layer, the content of fine particles such as silt and very fine sand decreased sharply, while the content of coarse sand and gravel rose rapidly. This may be due to sparse rainfall; the root systems of young saxaul plantations are still shallow and have limited soil retention capacity, leading to a lack of moisture in the deeper soil layers. As a result, fine particles cannot remain stable and are easily carried away by rain or wind, thereby disrupting the stability of the soil’s vertical structure and increasing the risk of soil desertification [38]. In Zone II, the vertical fluctuations in the content of silt, very fine sand, and fine sand in the 3-year-old saxaul plantations were relatively smaller than in Zone I. The contents of fine sand and very fine sand were also more stable. Under these precipitation conditions, saxaul plantations were better able to regulate soil particle distribution and improve soil quality. The decrease in gravel content within the plantations in Zone III was relatively small, indicating that saxaul plantations in areas with moderate precipitation cause less surface disturbance. This is consistent with the findings of previous studies, which suggest that vegetation restoration can stabilize particle size structure, particularly with smaller fluctuations in fine particle content in the vertical profile [39].

Significant differences were observed in localized soil nutrient contents between inside-shelterbelt near-plant positions and adjacent open bare ground across precipitation gradients. In Zone II, soil nutrient contents at inside-shelterbelt near-plant positions were higher than those in adjacent open bare ground, indicating localized nutrient enrichment under moderate precipitation conditions. This localized enrichment may be related to plant-associated processes, such as root exudates and litter return near saxaul individuals [40]. This pattern is consistent with previous research findings, which indicate that moderate water input enhances nutrient cycling efficiency, whereas both excessively low and high precipitation levels inhibit nutrient accumulation [41]. As plantation age increases, particularly in 6-year-old plantations, nutrient levels have shown a marked improvement, suggesting that vegetation restoration and root distribution play a positive role in soil nutrient accumulation. In Zone III, TN and TP concentrations peaked in the surface soil layer, suggesting that this precipitation level not only promotes the accumulation of available nutrients but also effectively enhances nutrient retention. In contrast, nutrient concentrations in the deeper 10–100 cm soil layer were more variable, as leaching caused by heavy rainfall weakened nutrient retention [42]. Although plantation age has a positive effect on surface soil nutrients, the difference in plantation age is less pronounced in deep soil layers. This may be because, in high-precipitation zones, the impact of nutrient loss outweighs that of plantation age, leading to unstable changes in soil nutrient levels. In contrast, in Zone I’s arid environment, wind erosion is significant, and overall soil nutrient levels are low, with no significant differences between inside- and outside-plantation soils. This indicates that water limitation is the primary factor restricting plant growth, which in turn affects soil nutrient accumulation.

Overall, the stability of the gravel layer and soil nutrient retention are not independent processes; rather, they are jointly regulated by precipitation conditions and together determine the sustainability of saxaul plantations in the Gobi region. In low-precipitation areas, once the gravel layer is disrupted by land preparation and afforestation activities, it easily triggers a chain reaction of surface roughening, fine-particle loss, and nutrient depletion. This not only makes it difficult for afforestation to generate stable ecological benefits but may also exacerbate the risk of soil degradation. Based on the findings of this study and management practices, it can be concluded that extremely arid Gobi regions are not suitable for large-scale saxaul plantations.

It should be noted that the inside-shelterbelt soil samples in this study were mainly collected near the base of saxaul plants. Therefore, changes in soil nutrients more likely reflect local soil responses within the plant-neighboring area and cannot be directly extrapolated to changes in the nutrient pool at the whole-plantation scale. Nutrient enrichment inside the shelterbelts may be related to plant interception, root activity, and the accumulation of fine particles, but its spatial extent and persistence still need to be further verified through more intensive spatial sampling.

### 4.3. Coupled Regulation Mechanisms of “Precipitation–Soil–Vegetation” Inside and Outside Plantations

This study used a partial least squares path modeling (PLS-PM) approach to reveal significant differences in the coupled relationships among precipitation, soil texture, soil fertility, and plant growth inside and outside plantations (Figure 7). Within the plantation, precipitation, soil texture, and soil fertility are key factors driving plant growth. Soil texture significantly promotes growth, while the effect of soil fertility is weaker, indicating that saxaul plantations directly improve water availability and root zone aeration by enhancing soil structure and optimizing particle size distribution, thereby enhancing plant growth capacity [43]. At the same time, soil texture has a significant negative impact on fertility, which may be related to the fact that an increase in fine-particle content is often accompanied by rapid uptake and turnover of available nutrients by roots and microorganisms [44].

Compared to the outside-plantation model, the effects of each pathway in the inside-plantation model were generally stronger and more significant; furthermore, the R^2^ for plant growth in the inside-plantation model was 0.51, indicating strong explanatory power and suggesting that plant growth plays a crucial role in the ecological stability of the plantation. This result is consistent with the understanding of the “resource island effect” and the coupling of wind-sand and hydrological processes in arid regions [45,46]. At the same time, the Gobi gravel layer also plays a significant role: an intact gravel layer can protect fine particles, reduce near-surface wind speeds and rain splash, and decrease evaporation; it also promotes surface water retention during light rain and dissipates kinetic energy during heavy rain to reduce fine-particle loss [47]. This makes it easier for soil available water and fine-particle nutrients to be retained in the root zone. This is consistent with the results of the inside-plantation model [48].

Conversely, common afforestation disturbances, such as large-scale pit preparation, trench and pipe excavation, and replanting and backfilling, tend to disrupt the interlocking and sorting of the gravel layer, leading to surface roughening, disruption of capillary continuity, and preferential flow. This results in rainfall infiltrating rather than being stored, and accelerates the loss of fine particles and available nutrients [49]. This is consistent with the weakened texture/fertility-to-growth pathways and reduced overall explanatory power observed in the outside-plantation model. Therefore, the PLS-PM model not only reveals the coupling relationships among rainfall, texture, and fertility but also provides mechanistic support for this study’s conclusions regarding the differentiation in the suitability of saxaul plantations in the Gobi region: In areas with moderate to high rainfall, saxaul plantations are more likely to form a positive feedback loop by improving soil texture and promoting the accumulation of surface resources; whereas in extremely arid regions, insufficient rainfall combined with disturbance of the gravel layer disrupts the positive “rainfall-texture-growth” coupling pathway, making it difficult for large-scale afforestation to achieve sustained and stable ecological benefits.

It should be noted that a small number of studies have reported that moderate gravel coverage can suppress evaporation and promote infiltration, thereby facilitating seedling establishment under specific scales and conditions, such as in plastic mulch-gravel coverage experiments in arid regions. This does not contradict the conclusions of this study; the differences primarily stem from variations in boundary conditions, including particle size and coverage, rainfall intensity and timing, plantation age, and vegetation density [50,51].

Correlation heatmaps further showed that the correlation structures of soil–vegetation variables differed between inside-shelterbelt and outside-shelterbelt samples. Inside the shelterbelts, plant growth indicators were more closely linked with soil particle-size composition and nutrient indicators, suggesting that after the establishment of artificial saxaul shelterbelts, vegetation growth may be jointly related to fine-particle accumulation, nutrient retention, and local microenvironment improvement. In contrast, although soil texture and nutrient indicators were related to some extent outside the shelterbelts, their correspondence with plant growth indicators was relatively weak, more likely reflecting background soil differences among precipitation zones. This result is consistent with the higher explanatory power of the inside-shelterbelt PLS-PM than that of the outside-shelterbelt model, indicating that tighter soil–vegetation coupling may have formed inside saxaul shelterbelts. It should be noted that both the correlation heatmaps and PLS-PM were based on field observational data. The model paths reflect directional relationships and model settings among variables under the precipitation gradient, rather than strict geomorphological causal evidence. Soil texture in the Alxa Gobi may still be jointly affected by parent material, long-term pedogenic processes, and geomorphic background. Therefore, the “precipitation gradient–soil–vegetation” relationship in this study is interpreted as variable coupling, rather than evidence that contemporary precipitation directly determines soil texture formation.

### 4.4. Management Implications for Saxaul Plantations in the Gobi Desert Under Ecological Feasibility and Cost-Effectiveness Constraints

In addition to ecological constraints, the mismatch between afforestation costs and benefits further illustrates that the blind expansion of saxaul plantations in extremely arid Gobi regions lacks sustainability. Taking the Alxa region as an example, the three-year afforestation costs for one-year-old saxaul shelterbelts reached 11,169.41, 11,641, and 9832.29 yuan·hm^−2^ in Zuoqi, Youqi, and Ejinaqi, respectively, all exceeding the national standard of 6000–11,000 yuan·hm^−2^ [15]. In low-precipitation Gobi regions (≤50 mm), the cost per unit area of successful stand establishment often rises further due to low survival rates, high frequency of replanting and maintenance, and substantial investment in surface protection facilities, while the corresponding ecological benefits are difficult to be realized consistently in the short term. At the same time, excessively high initial planting density increases seedling costs and subsequent replanting expenses, while intensifying competition among individuals for limited water resources, thereby further reducing the efficiency of successful stand establishment. In contrast, in areas with medium to high precipitation, particularly within the 50–100 mm range, saxaul plantations are more likely to achieve higher survival rates and better growth conditions, enabling the formation of ecological barriers and soil improvement effects with relatively lower risk. However, the results of this study also indicate that even in relatively suitable precipitation zones, survival rates are still significantly influenced by variations in initial planting density; excessively high densities can undermine the establishment advantages brought about by improved precipitation conditions. Therefore, from the dual perspectives of ecological feasibility and economic rationality, extremely arid Gobi regions do not possess the appropriate conditions for large-scale promotion of saxaul plantations.

**Figure 9:**
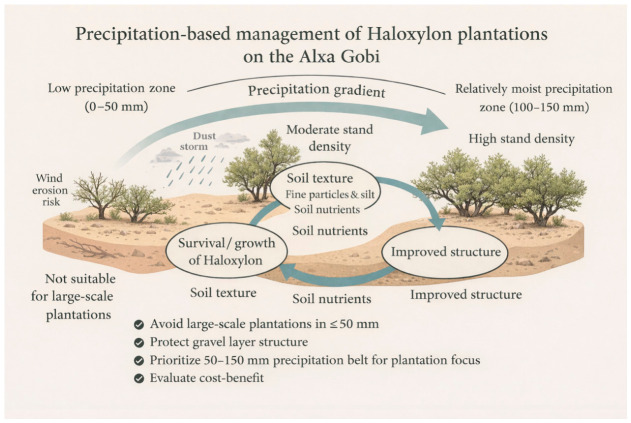
Precipitation-based management of *saxaul* plantations on the Alxa Gobi. Note: The green aboveground parts of saxaul represent green assimilating branches rather than true leaves.

Based on the findings of this study and the lessons learned from existing large-scale ecological projects, it can be concluded that precipitation conditions should serve as a key factor in determining the spatial planning of saxaul plantations in the Gobi region. Cao et al. [33] noted that projects such as China’s Three-North Shelterbelt Program commonly face issues including low survival rates, poor growth, and soil moisture depletion in areas with annual precipitation < 250 mm. This study further indicates that, in Gobi environments, approximately 50 mm can be considered the practical lower limit for artificial saxaul establishment. Accordingly, saxaul plantations in the Gobi region can be divided into three management zones: in areas with ≤50 mm, large-scale afforestation should be avoided, with a focus on low-disturbance ecological conservation and natural restoration; in areas with 50–100 mm, conditional afforestation may be carried out under the strict control of initial planting density, optimization of stand configuration, and enhanced site selection to avoid a decline in survival rates due to excessive density; areas with 100–150 mm rainfall may be designated as relatively priority zones, but must still adhere to the principles of “greening based on water availability” and “the right species for the right site.” Uniform expansion and homogeneous implementation should be avoided, and planting should be adjusted to site conditions through moderate densification or medium-to-low density configurations, rather than simply pursuing high-density afforestation. Based on the above, this study proposes a precipitation-suitability management framework for saxaul plantations in the Alxa Gobi (Figure 9). Furthermore, in terms of specific site selection, priority should be given to areas with intense sand hazard activity, ecologically fragile zones at the edges of oases, and areas around salt lakes or in saline–alkali habitats where water and salinity conditions are relatively moderate, groundwater conditions are relatively stable, and there is a need for ecological protection. This will fully leverage the practical benefits of saxaul plantations in windbreak and sand fixation, oasis protection, and the construction of ecological barriers.

At the same time, protection of the gravel layer should be a key component of saxaul plantations management in the Gobi region. This study indicates that afforestation disturbances in low-precipitation areas can easily damage the surface gravel layer, triggering a chain reaction of surface coarsening, fine-particle loss, and nutrient depletion; even in medium- to high-precipitation areas, improper land preparation methods may weaken the positive feedback between soil structure improvement and nutrient accumulation. Furthermore, excessively high initial planting density may intensify intraspecific competition, thereby exacerbating water consumption pressures and resource allocation conflicts following surface disturbance, which is detrimental to the long-term stability of the stand. Therefore, in areas with well-developed gravel layers, destructive afforestation methods such as large-pit site preparation, deep plowing, and extensive surface disturbance should be avoided. Instead, low-disturbance measures such as small-hole planting and narrow-slot planting should be prioritized. Where necessary, the native gravel layer should be retained or restored outside the planting pit to maintain surface structural stability and reduce the risk of wind erosion. Furthermore, density regulation should be a key component of afforestation design. Appropriate initial planting densities should be determined based on precipitation conditions, soil texture, and the condition of the gravel layer, avoiding the practice of using high-density afforestation to achieve short-term canopy cover. Prior to afforestation, assessments of soil, gravel layers, and site conditions should be conducted. Precipitation conditions, gravel layer integrity, initial planting density plans, and cost–benefit analyses should be incorporated into the project approval and layout optimization processes to enhance the scientific rigor and sustainability of afforestation decisions in the Gobi region.

## 5. Conclusions

This study shows that precipitation is a key factor controlling the establishment success of artificial saxaul shelterbelts in the Alxa Gobi. Overall, saxaul survival rate and growth indicators increased with increasing precipitation and plantation age, and an annual precipitation of approximately 50 mm can be regarded as a practical lower limit for large-scale establishment of artificial saxaul shelterbelts in Gobi regions. In extremely low-precipitation areas with annual precipitation ≤ 50 mm, saxaul survival and growth were clearly constrained, and afforestation disturbance may damage the surface gravel layer, leading to soil coarsening and intensified wind erosion. In contrast, in areas with 50–150 mm precipitation, saxaul shelterbelts with appropriate planting density are more conducive to the accumulation of fine particles and nutrients, thereby forming relatively favorable gravel–soil feedbacks. Precipitation gradient, soil texture, soil fertility, and saxaul growth were closely coupled, and this coupling was stronger inside the shelterbelts than outside the shelterbelts. Overall, future saxaul afforestation in extremely arid Gobi regions should comprehensively consider precipitation conditions, gravel-layer integrity, appropriate planting density, and the balance between afforestation costs and benefits, so as to improve establishment success and maintain favorable soil feedback processes and sustainability.

## Figures and Tables

**Table 1:** Climatic Characteristics Across Different Precipitation Gradients in the Study Area.

Precipitation Zone	Mean Annual Precipitation (mm)	Mean Annual Temperature (°C)	Mean Annual Evaporation (mm)	Mean Annual Wind Speed (m·s^−1^)
Zone I (0–50 mm)	32.8 ± 12.4	8.9 ± 0.8	3840 ± 320	4.2 ± 0.6
Zone II (50–100 mm)	105.2 ± 15.2	8.8 ± 0.7	3900 ± 280	3.9 ± 0.5
Zone III (100–150 mm)	132.6 ± 28.9	7.2 ± 0.6	3100 ± 200	3.1 ± 0.4

Note: Compiled based on data from the meteorological stations corresponding to the study areas.

**Table 2:** Planting density and survival rate in sample plots of *saxaul* under different precipitation gradients and plantation ages.

Precipitation (mm)	Plantation Age (Years)	Number of Plots	Planting Density (Plants·ha^−1^)	Survival Rate (%)
0~50	2a	4	574 ± 65	0.27 ± 0.12
	6a	6	502 ± 60	0.54 ± 0.12
50~100	3a	10	1392 ± 406	0.54 ± 0.26
	4a	18	1160 ± 240	0.81 ± 0.22
	6a	3	2087 ± 257	0.57 ± 0.01
100~150	4a	4	1710 ± 460	0.51 ± 0.09
	6a	3	1346 ± 553	0.68 ± 0.23

**Table 3:** Comparison of growth parameters of *saxaul* under different site types and plantation ages.

Site Type	Soil Characteristics	Plantation Age (a)	Survival Rate	Plant Height (m)	Canopy Area (m^2^)	Basal Diameter (mm)	References
Saline Lake/Salinized Habitat	Characterized by some degree of salinization; salt content is higher than that in typical desert habitats, and fine-particle content is higher than that in the Gobi.	2–3	0.97	0.76 ± 0.11	0.57 ± 0.16	2.63 ± 0.15	[34,35,36]
		5–6	0.95	1.05 ± 0.12	1.30 ± 0.20	3.27 ± 0.25	
		9–10	0.93	1.26 ± 0.07	1.80 ± 0.13	4.63 ± 0.42	
Desert (fixed/semi-fixed dunes)	High fine-particle content, loose soil texture, and good aeration; nutrients tend to accumulate in the topsoil.	2–3	0.84	0.8 ± 0.12	0.41 ± 0.05	1.6 ± 0.16	
		5–6	0.80	1.27 ± 0.09	0.88 ± 0.09	4.0 ± 0.31	
		9–10	0.78	2.60 ± 0.09	1.06 ± 0.12	6.3 ± 0.66	

## Data Availability

The raw data supporting the conclusions of this article will be made available by the authors on request.
